# Analysis on professional identity and related factors among Chinese general practitioners: a National Cross-sectional Study

**DOI:** 10.1186/s12875-020-01155-4

**Published:** 2020-05-06

**Authors:** Liqing Li, Yong Gan, Yudi Yang, Heng Jiang, Kai Lu, Xiaogang Zhou, Zhiqiang Nie, Sampson Opoku, Yanling Zheng, Fang Yu, Zuxun Lu

**Affiliations:** 1 Department of Management Science and Engineering, School of Economics and Management, Jiangxi Science and Technology Normal University, Nanchang, Jiangxi China; 2 Department of Social Medicine and Health Management, School of Public Health, Tongji Medical College, Huazhong University of Science and Technology, No. 13 Hangkong Road, Wuhan, 430030 China; 3 Centre for Alcohol Policy Research, School of Psychology and Public Health, La Trobe University, Melbourne, Victoria Australia; 4 Melbourne School of Population and Global Health, University of Melbourne, Melbourne, Victoria Australia; 5 Office of Student Affairs, Tongji Hospital, Tongji Medical College, Huazhong University of Science and Technology, Wuhan, Hubei China; 6 School of Economics and Management, East China JiaoTong University, Nanchang, Jiangxi China; 7 Shouyilu Street Community Health Service Center of Wuchang District, Wuhan, Hubei China

**Keywords:** General practice, Professional identity, Influencing factors, Primary care, China

## Abstract

**Background:**

Studies on professional identity and related factors among Chinese general practitioners (GPs) are unavailable. The objective of this study was to investigate the professional identity level of GPs in China and explore factors associated with GPs’ perceptions of their professional identity.

**Methods:**

A multistage stratified random sampling method was used to collect data with a structured self-administered questionnaire from 3236 GPs working in community health service institutions (CHIs) in China between October, 2017 and February, 2018. Professional identity was measured by the 13 items scale. Descriptive statistics were calculated and groups’ differences were estimated using nonparametric tests. Multiple linear stepwise regression analysis was used to analyze factors associated with professional identity among GPs.

**Results:**

Based on a total score of 65 on the professional identity scale, the average score for GPs’ professional identity was 51.23 (SD = 6.56). Multiple linear stepwise regression analysis showed that GPs who practiced in Central China, with an administrative responsibility, at a moderate or higher income level, who frequently worked overtime, had more occupational development opportunities, with a higher level of job satisfaction and older GPs had higher levels of professional identity.

**Conclusions:**

Professional identity level among GPs in China is high. Region, administrative responsibility, income level, working overtime, occupational development opportunities, age, and job satisfaction were significant predictors of professional identity.

## Background

General practitioners (GPs) are the main providers of community health service (CHS). They provide a wide range of healthcare services to residents [1]. Shortage of GPs and high turnover have been seen as frequently reported problems in both developed and developing countries. The GP workforce in the United Kingdom (UK) is insufficient to meet current demand [2]. In addition, there is a dramatic shortage of GPs in Germany, especially in rural areas [3], and more than half of physicians (57%) contemplated leaving general practice in New Zealand [4]. Among key steps aimed at addressing these shortages include increases in recruitment and retention of GPs and to greater user of other health professionals [5]. In spite of these strategies, it has been difficult to recruit doctors as GPs in adequate numbers in the UK [6, 7]. Other countries also have difficulties in recruiting enough GPs [8–10]. The Finnish healthcare system is faced with similar challenges with GPs in addition to high turnover among primary care physician [11]. The quantity and quality of GPs workforce are related to the effectiveness and quality of health care delivery [12], thus, shortage of GPs in the CHS of China threatens the provision of quality health care.

Professional identity is often defined as career, occupational or even vocational identity [13]. It is a component of people overall identity and is augmented by the ‘position within society’, ‘interactions with others’ and their ‘interpretations of experiences’ [14]. The perception of professional identity varies among individuals, as experience and understanding are different among practitioners [15]. Professional identity has been identified as a key factor in GPs’ ability to provide high-quality care to improve patient outcomes [16].

GPs constitute an important part of China’s primary health care workforce, and their shortage has been identified as an urgent priority that should be addressed [17]. A positive and flexible professional identity is significant for GPs to enable them function at a high level since their performance benefits not only themselves, but also patients and other healthcare workers [18]. Professional identity is inextricably linked to, yet separated from overall self-concept [19] and can be measured and actively cultivated [20]. Previous studies have been conducted to examine professional identity and its associated factors (e.g., sex [21], age [22], education experience [18], job satisfaction [23], doctor-patient relationship [23], and turnover intention [17]) among nurses. Other studies have also been conducted to examine professional identity among specific healthcare workers such as township health inspectors [17], registered nurses [18, 24], physicians [23], midwives [25], nursing students [26], graduate nursing students [21], telephone GPs (GPs working on the helpline) [27], and prison health workers [28]. However, no study has been conducted to examine the status of professional identity and its determinants among GPs. Therefore, this study aimed to investigate professional identity and related factors among GPs in China.

## Methods

### Study population

A cross-sectional study was conducted among Community health service institutions (CHIs) in China from October 2017 to February 2018. The study sample was obtained by a multistage stratified random sampling procedure. In the first stage, 4 provinces or municipalities from each of eastern (Shanghai, Beijing, Guangdong, and Zhejiang), central (Hubei, Anhui, Heilongjiang, and Heinan) and western regions of China (Sichuan, Chongqing, Guizhou, and Yunan) were randomly selected. In the second stage, 30 CHIs were randomly selected from each province. In the third phase, according to the number of GPs and scales of CHIs, 40% of the in-service GPs with at least 1 year of work experience were randomly selected to complete a self-administered questionnaire. A total of 3244 GPs were asked to complete the self-reported questionnaire via WeChat, of which no response was received from 8 GPs. Finally, a total of 3236 GPs finished and returned the questionnaire.

This study was approved by the ethics committee of Tongji Medical College Institutional Review Board, Huazhong University of Science and Technology, Wuhan, China. Written informed consent was obtained from all survey participants.

### Instrument and measurement

The questionnaire comprised two sections. The first section collected information on region, age, sex, education level, management responsibility, marital status, professional title, contract status, income level, working overtime, occupational development opportunities, job satisfaction, and work stress. The second section comprised a13-item scale regarding professional identity. Professional identity in this study was assessed by a modified version of the 13-item professional identity assessment scale. The Chinese version of this scale had been modified according to studies by Zhao [29] and Wu [30]. These 13 items were assessed on a five-point Likert scale, ranging from 1(strongly disagree) to 5 (strongly agree) (Table 1). The total scores ranged from 13 to 65, with higher scores indicating higher professional identity among GPs. Scores 13–17 indicated low level of professional identity; 18 to 33 indicated medium level of professional identity; ≥34 indicated high level of professional identity.

**Table 1:** Professional Identity assessment Scale (*n* = 3236)

Items	***Level of professional identity***				
	Strongly disagree n (%)	Somewhat disagree n (%)	Neutral n (%)	Somewhat agree n (%)	Strongly agree n (%)
1. When referring to my profession, I usually say “we” rather than “they”.	63 (1.9)	132 (4.1)	447 (13.8)	1949 (60.2)	645 (19.9)
2. I consider my success as the success of health care workers.	89 (2.8)	303 (9.4)	734 (22.7)	1615 (49.9)	495 (15.3)
3. I care very much about other people’s views on my career.	73 (2.3)	321 (9.9)	648 (20.0)	1715 (53.0)	479 (14.8)
4. The praise my profession from the people, which = is the praise they give on me.	72 (2.2)	254 (7.8)	565 (17.5)	1705 (52.7)	640 (19.8)
5. If there are some criticisms on my career from the media, I will feel ashamed and embarrassed.	126 (3.9)	305 (9.4)	458 (14.2)	1579 (48.8)	768 (23.7)
6. My job is important. /I believe in the importance of my job.	36 (1.1)	96 (3.0)	393 (12.1)	1767 (54.6)	944 (29.2)
7. I am confident in my ability on work.	27 (0.8)	45 (1.4)	323 (10.0)	1968 (60.8)	873 (27.0)
8. My job will exercise effects on patients’ conditions.	121 (3.7)	190 (5.9)	524 (16.2)	1801 (55.7)	600 (18.5)
9. My job is meaningful.	30 (0.9)	81 (2.5)	357 (11.0)	1809 (55.9)	959 (29.6)
10. I have the necessary qualifications and skills for my job.	22 (0.7)	53 (1.6)	322 (10.0)	2075 (64.1)	764 (23.6)
11. I understand the responsibilities and requirements of the work.	20 (0.6)	24 (0.7)	205 (6.3)	2149 (66.4)	838 (25.9)
12. Healthcare work suits me.	31 (1.0)	100 (3.1)	564 (17.4)	1866 (57.7)	657 (20.3)
13. I know my role.	24 (0.7)	35 (1.1)	314 (9.7)	2113 (65.3)	750 (23.2)

In this study, a Greatest Lower Bound (GLB) method [31] was used to estimate the internal consistency of the Likert scale scoring to see whether they all reliably assessed professional identity. The GLB and GLB algebraic (GLBa) coefficients for the scale were 0.931 and 0.940, respectively. This indicated a good internal consistency. Bartlett’s test of sphericity, which tested the overall significance of all the correlations within the correlation matrix, was significant (*χ*^2^ (78) =21,718.23, *P* < 0.001), indicating that it was appropriate to use the factor analytic model on this set of data.

### Data collection and quality control

We designed a questionnaire based on literature review, group discussions and mock interviews. To improve the quality of questionnaires, a pre-test was done at Wuhan’s CHIs. A web-link to the online questionnaire was designed with a tool of Questionnaire Star Software and then disseminated to GPs via WeChat. The data were entered into the Web-based database by specialized investigators to ensure accuracy.

### Statistical analysis

Professional identity was the dependent variable. In the multivariable model, predictive variables included age, region, sex, marital status, education level, contract status, management responsibility, working overtime, income level, professional title, work stress, occupational development opportunities, and job satisfaction. Multicollinearity was assessed with the variance inflation factor [32].

STATA version 12.0 was used for database assembling and statistical analysis. The distribution of the data was assessed. Categorical variables will be presented as numbers and percentages and descriptive statistics of professional identity of GPs will be presented across categorical variables. In this study, because the data distribution of professional identity was non-normal (W-statistics = 0.940, *P* < 0.0001), the nonparametric tests (Kruskal-Wallis test and Wilcoxon test) were used to compare the difference across groups. We used multiple linear stepwise regression analysis to explore the factors associated with professional identity among GPs. A value of *P* < 0.05 (two-tailed) was considered statistically significant.

## Result

The results from the professional identity scale across respondents and items are shown in Table 1. Among these 3236 GPs (response rate, 99.75%), 15 (0.5%), 33(1.0%), 3188 (98.5%) of them had a low level of professional identity, medium level of professional identity, and high level of professional identity, respectively. The average score for GPs’ professional identity was 51.23 (SD = 7.06).

Table 2 presents the main characteristics of respondents. A total of 3, 236 participants were involved in the study, and the ages ranged from 21 to 80 years (Mean = 37.42, SD = 7.92). Among these participants, 37.98, 30.01, and 32.01% were from Eastern China, Central China and Western China, respectively. More than half (63.84%) of them were females. The majority of GPs (85.63%) were married. More than 65 % (66.10%) of the study participants had a bachelor’s degree, and 67.52% had permanent work contracts. Less than a third of them (24.17%) had management responsibilities. Few respondents (5.62%) were not doing overtime duties on holidays or weekends. Overall, most of GPs (70.49%) were at moderate or lower-income level. More than half (58.56 and 51.55%) of GPs reported higher level of work stress and higher level of job satisfaction, respectively. Few participants (7.32%) had occupational development opportunities.

**Table 2:** Descriptive statistics for determinates and associations with professional identity of GPs in China (n = 3236)

Variable		Frequency (%)	Median (IQR)	***Z value***	***P*** value
Region	Eastern China	1229 (37.98)	51 (6)	11.92	0.003
	Central China	971 (30.01)	52 (8)		
	Western China	1036 (32.01)	51 (7)		
Sex	Male	1170 (36.16)	52 (7)	2.15	0.03
	Female	2066 (63.84)	51 (6)		
Marital status	Unmarried/widow/divorced	465 (14.37)	51 (6)	−2.89	0.004
	Married	2771 (85.63)	52 (7)		
Education level	Associate’s degree or vocational diploma^a^	918 (28.37)	52 (6)	0.18	0.91
	Bachelor degree	2139 (66.10)	51 (7)		
	Master degree or higher	179 (5.53)	51 (9)		
Contract status	Permanent^b^	2185 (67.52)	51 (7)	0.18	0.86
	Temporary	1051 (24.38)	52 (7)		
Management responsibility	Yes	782 (24.17)	52 (7)	5.81	< 0.0001
	No	2454 (75.83)	51 (6)		
Working overtime	Never	182 (5.62)	52 (7)	9.95	0.007
	Occasion	1759 (54.36)	51 (6)		
	Frequent	1295 (40.02)	52 (8)		
Income level	Moderate or below	2281 (70.49)	51 (7)	64.50	< 0.0001
	Moderate	864 (26.70)	52 (7)		
	Moderate or higher	91 (2.81)	54 (11)		
Professional title	Elementary or below	1419 (43.85)	51 (6)	14.59	< 0.0001.
	Intermediate	1412 (43.63)	52 (7)		
	Senior	405 (12.52)	52 (7)		
Work stress	Low	313 (9.67)	52 (7)	5.297	0.07
	Intermediate	1028 (31.77)	52 (6)		
	High	1895 (58.56)	51 (7)		
Occupational development opportunities	Fewer	1650 (50.99)	51 (7)	90.17	< 0.0001
	General	1349 (41.69)	52 (6)		
	More	237 (7.32)	53 (8)		
Job satisfaction	Low	1220 (37.70)	50 (7)	228.47	< 0.0001
	Medium	348 (10.75)	50 (6)		
	High	1668 (51.55)	52 (6)		
Age (years)	21-	515 (15.91)	51 (6)	20.51	< 0.0001
	30-	1454 (44.93)	51 (7)		
	40-	1063 (32.85)	52 (7)		
	50-	204 (6.30)	52 (6.5)		
Work tenure (years)	1-	2241 (69.25)	51 (7)	3.573	0.17
	10-	825 (25.49)	52 (7)		
	20-	170 (5.25)	52 (5)		

*IQR* interquartile range

^a^ GPs who have acquired associate degree or vocational diploma. An associate degree required 3 years of education in college after graduation from senior middle school (grade year 10 to year 12), or 5 years of education in college after graduation from junior middle school (grade year 7 to year 9). A vocational diploma requires 2 years of education in vocational schools after graduation from senior middle school, or 3 years of education in vocational schools after graduation from junior middle school

^b^Wages and welfares of GPs would be paid by the Chinese government public health services expenditure. GPs could practice in the health institutions to retire

Table 2 demonstrates results comparing the differences in median scores of professional identity among various groups. There were significant differences in GPs’ professional identity across regions, sex, marital status, management responsibility, working overtime, income level, professional title, occupational development opportunities and job satisfaction (*P* < 0.05). There were no significant differences in professional identity among GPs with regard to education level, contract status, and work stress (*P* > 0.05).

Table 3 shows results from the multiple linear stepwise regression analysis to determine factors associated with GPs’ professional identity. Region, management responsibility, income level, working overtime, occupational development opportunities, age, and job satisfaction were significantly related to professional identity. GPs from central China, those who had management responsibility, who were at a moderate or higher income level, who often worked overtime, who had more occupational development opportunities, who had higher job satisfaction and were older had higher professional identity. There was no multicollinearity in this study since the variance inflation factors for these variables were below the value of 2.1.

**Table 3:** Multivariable stepwise linear model analysis for the association with the professional identity among GP (N = 3236)^a^

Variables (reference)	***β***	***SE***	***t***	***P value***
**Region (Central China)**				
Eastern China	−1.18	0.29	−4.08	0.001
Western China	−0.67	0.30	−2.22	0.03
**Management responsibility (No)**				
Yes	0.73	0.29	2.52	0.01
**Income level (Moderate or below)**				
Moderate or higher	1.64	0.67	2.44	0.02
**Working overtime (Never)**				
Frequent	1.12	0.25	4.53	< 0.0001
**Occupational development opportunities (Fewer)**				
More	1.35	0.47	2.85	0.004
**Age** (per 10 years)	0.5	0.02	3.08	0.002
**Job satisfaction**	0.33	0.02	17.23	< 0.0001
**Intercept**	37.84	0.91	41.47	< 0.0001

*R*^2^ = 0.1129, Adj *R*^2^ = 0.1107; *F* = 51.35, *P* < 0.0001

^a^Adjustment for sex (female and male), education level (associate’s degree or vocational diploma, bachelor degree, master degree or higher), marital status (unmarried or widowed or divorced, married), professional title (elementary or below, intermediate, senior), contract status (permanent, temporary), work stress (low, intermediate, high), work tenure (continuous), and other variables in the models

## Discussion

The demand for primary healthcare services amongst the Chinese population is increasing and GPs constitute an essential part of the human resources need to provide these services. Training and retention of GPs are prudent strategies to improve quality care outcomes. Improvement in GPs’ professional identity is important for the provision of high-quality patient care. Identifying factors associated with professional identity would help Chinese government to implement effective plans to strengthen the GPs workforce.

This study investigated professional identity and related factors among GPs in China. It showed that Chinese GPs had a high level of professional identity. Further, our findings showed that significant factors associated with professional identity included age, region, management responsibility, income level, working overtime, occupational development opportunities, and job satisfaction.

The Chinese version of the 13-item professional identity assessment scale has been used among Chinese healthcare workers, which was different from the versions commonly used in western countries [33–36]. For example, Cowin et al. [35] in 2013 showed that professional identity results from the internalization of professional knowledge, skills, attitudes, values and standards of ethics, and then the integration of these characteristics into one’s personal identity and behaviors within practice. Another study conducted by Deppoliti [36] reported that professional identity is the person’s perception of himself/herself as the member of the profession, which has a core meaning of occupational integration. By contrast, our professional identity has more focused on the commitment and importance of the profession itself. This scale has paid more attention to the health care workers’ views and attitudes towards social value of profession, and which was more suitable within Chinese culture and health system.

This study showed that there was a statistically significant relationship between professional identity and age (*β =* 0.5). Previous studies have found that there was a slightly positive correlation between age and professional identity [37–40]. Again, in terms of age, two earlier studies [22, 41] concluded that with increasing age whilst working, GPs acquire more experience in dealing with doctor-patient relationships and communications, patient outcomes and the environment resulting in the acquisition of more professional and technical skills compared to younger colleagues, thereby helping them to improve their professional identity.

GPs from Central China recorded the highest professional identity compared to those from eastern (*β =* − 1.18) and western regions of China (*β =* − 0.67). It is known that, Eastern China developed more rapidly than Central and Western China because of the geographical advantage. After the eastern priority development strategy, the Chinese government formulated a balanced development strategy. With the policy of raising Central China, the local government invested massively in education, economy, healthcare, etc. Compared with GPs practicing in Western China, Central China GPs had more advantages with respect to income and occupational development opportunities, which led to improvement in their professional identity levels at work.

GPs who had management responsibility reported a higher level of professional identity than those without (*β =* 0.73). One possible interpretation could be that, GPs who had management responsibility were more likely to be satisfied with their current work and were likely to feel a sense of accomplishment from their job. This personal value that ensured in their work was an important factor for professional identity. Another possible explanation was that those GPs with more professional identity aspire to, or are chosen for management positions. A higher level of professional identity among GPs would lead to higher commitment and greater passion to their work; they may be pleasured to undertake an administrative or management responsibility.

According to our results, professional identity of respondents at moderate or higher income level were higher than those at moderate or lower income level (*β =* 1.64), which indicated that income level was significantly related to professional identity. This finding was comparable to an observation conducted in Taiwan, where nurses’ wages were positively correlated with professional commitment [24]. Increasing income levels could be an effective way to improve the professional identity of GPs. In Sweden, the UK, and the United States, GPs’ income was 2 ~ 4 times higher than their social average income [42, 43]. However, in China, the income of GPs was less than average income and far less than specialists’ income, especially among those working in primary heath institutions.

The more overtime work GPs do, the higher their professional identity level. The professional identity level of GPs with frequent overtime works was higher than those with no overtime work (*β* = 1.12). One possible reason is that there is a positive relationship between working overtime and a feeling of pride and personal achievement, because healthcare professionals belong to a noble profession. Traditional Chinese culture considers doctors as saviors. GPs interviewed described their professional identity using the traditional Chinese idiom: reviving the dying and curing the wounded [23], which is consistent with traditional Chinese culture [44]. On the contrary, GPs, who have a higher level of professional identity, maybe have a frequent overtime work in their practices. When some aspects of an individual’s identity are consistent with his occupation, individuals would keep a high level of work engagement even in unfavorable work environments [45]; thus, they may have a frequent overtime work in their career.

The results of our research also showed that higher job satisfaction may lead to higher professional identity (*β* = 0.33). Studies conducted in Taiwan [24] and Turkey [33] revealed that there was a positive correlation between job satisfaction and professional identity among nurses. Thus, improvement in job satisfaction can be an important tool to increase professional identity among GPs. In addition, previous studies [17, 33, 46] showed that the professional identity was an important predictor of job satisfaction. When individuals have a positive identity with their careers, they will devote more energy and enthusiasm to their work, and the dissatisfaction resulted from working environment would be eliminated to a certain extent [47].

The results of our study indicated that participants with occupational development opportunities had a higher level of professional identity than those without occupational development opportunities (*β* = 1.35). The occupational development opportunity was designed to clearly spell out the roles and responsibilities of GPs in an organization, which helped to establish their professional identity. Previous studies have shown that continuous professional development may influence the perceptions on professional identity [48, 49]. Fewer occupational development opportunities seemed to drive GPs away from primary health care. However, more occupational development opportunities can help attract more GPs. A previous study supported our finding showed that lack of or inadequate occupational development opportunities on a job may reduce efficiency, cut or reduce enthusiasm or even lead to staff resignation [50].

Notably, values of professional identity might be biased by social desirability, which was very common in psychology and the social sciences [51, 52]. Some of the methodological remedies that can be employed to reduce bias include question randomization, anonymity and confidentiality, double-blind protocols, and grouped answer method [51]. In the present study, we have used the anonymous and web-based survey administration to collect data. Compared with in-person or phone-based administration, this method has been shown to elicit higher reporting of items with social desirability bias [53].

### Strengths and limitations

This study has several strengths. Firstly, this is the first study with a nationally representative sample of Chinese GPs to investigate professional identity and associated factors in China. Secondly, the present study had a large sample and captured the target group in CHS of Eastern China, Central China, and Western China. The large sample size significantly increased statistical power to identify social determinates of professional identity among GPs. Thirdly, with the popularity of smart phones and rapid development of communication tools in China, a web-based survey approach was employed in our study. The study was conducted on a free platform and could minimize missing data because participants would not be able to proceed to the next question unless the required data field is filled.

However, some limitations of our study should be acknowledged. Firstly, the potential influencing factors of GPs’ professional identity could be more than those included in the questionnaire. Secondly, this was a cross-sectional study design, which precluded evaluation of temporality and causality of the observed relationships. Thirdly, small beta values were observed in our study, which may be related to the sample size. Additional studies with larger sample size are warranted to investigate factors associated with professional identity among GPs.

### Implications for research and practice

These findings indicate further studies with more potential factors related to professional identity are warranted. Health policy makers and think-tanks might take corresponding measures and strategies to enhance the professional identity of GPs. Actions such as offering higher and more rational income, providing more occupational development opportunities, and improving higher job satisfaction are needed.

## Conclusion

In summary, Chinese GPs have higher levels of professional identity. Age, region, management responsibility, income level, working overtime, occupational development opportunities, and job satisfaction are significantly associated with professional identity. The results of our study suggest that policymakers need to better understand the predictors of professional identity among GPs, and the Chinese government should take some measures to boost GPs’ professional identity to sustain the stability of GPs group.

## Supplementary information

**Additional file 1.**

## Data Availability

Data may be made available by contacting the corresponding author.

## Abbreviations

CHI: Community health service institution
CHS: community health service
GLB: greatest lower bound
GLBa: greatest lower bound algebraic
GP: general practitioner
