# Supplementary material for: Analysis on professional identity and related factors among Chinese general practitioners: a National Cross-sectional Study
Source: BMC Fam Pract. 2020 May 6;21:80. doi: 10.1186/s12875-020-01155-4 (PMC7201728; doi:10.1186/s12875-020-01155-4)
Supplement: Supplementary file 1 — Additional file 1. [file 12875_2020_1155_MOESM1_ESM.doc]

**全科医生职业吸引力调查表**

您工作单位所在地： 省 市 区/县

尊敬的全科医生：

您好！受中国社区卫生协会委托，我们正在开展一项有关全科医生职业吸引力的调查研究，为制订相关政策提供依据。请您根据个人情况、认识和体验如实填写问卷。我们采取匿名填表的方式，对您所填写的问卷我们严格保密，且只用于研究所用，请您放心填写。感谢您的支持与配合！

湖北省社区卫生服务中心主任联盟

中国社区卫生协会课题组

华中科技大学同济医学院公共卫生学院

# 一、基本情况

1、您的年龄： 岁

2、您的性别： 1）男 2）女

3、民族： 1）汉族 2）少数民族

4、婚姻状况： 1）未婚 2）已婚 3）离异 4）丧偶

5、您的最高学历是： 1）中专及以下 2）大专 3）本科 4）硕士 5）博士

6、您目前拥有的医疗保障制度是：（可多选）

1）自费 2）城镇职工医疗保险 3）城镇居民医疗保险 4）公费医疗 5） 新型农村合作医疗 6）商业医疗保险 7）其他

7、您从事医疗卫生工作的年限： 年：您在社区卫生服务中心工作的年限：____年，您从事全科医生工作的年限：_____年；您接受的全科医生资格培训（可多选）：1）岗位培训 2）骨干培训 3）转岗培训 4）规范化培训（三年）

8、您的任职方式： 1）正式在编 2）合同制 3) 临聘 4）返聘

9、您的职称： 1）无职称 2）初级 3）中级 4）副高 5）正高

10、您是否有行政职务： 1）有 2）无

11、您平时晚上或者节假日加班吗？

①不加班 ②偶尔加班 ③经常加班

12、最近一段时间，您在工作中是否有感到压力？

①无任何压力 ②压力较小 ③一般 ④压力较大 ⑤压力非常大

13、您感觉在本单位职业发展（晋升、提拔等）的机会如何？

①很少 ②较少 ③一般 ④较多 ⑤很多

# 二、工作满意度

| **说明：**这部分问卷目的在于了解您个人工作感受的相关资料。本部分每项陈述后有5个选项，用数字1-5表示从“非常不满意”到“非常满意”，请您在相应数字上打“√”。 | 非常不满意 | 不满意 | 一般 | 满意 | 非常满意 |  |
| --- | --- | --- | --- | --- | --- | --- |
| 1、您对您目前的收入状况满意吗? | 1 | 2 | 3 | 4 | 5 | |
| 2、您对单位提供的福利情况满意吗? | 1 | 2 | 3 | 4 | 5 | |
| 3、您对这里的生活环境满意吗? | 1 | 2 | 3 | 4 | 5 | |
| 4、您对单位的工作条件满意吗? | 1 | 2 | 3 | 4 | 5 | |
| 5、您对单位总体上的管理制度满意吗? | 1 | 2 | 3 | 4 | 5 | |
| 6、您对单位给您安排的工作量满意吗? | 1 | 2 | 3 | 4 | 5 | |
| 7、您对单位内部的人际关系满意吗? | 1 | 2 | 3 | 4 | 5 | |
| 8、您对当地居民对您工作的尊重和认可情况满意吗? | 1 | 2 | 3 | 4 | 5 | |
| 9、您对获得的培训机会满意吗? | 1 | 2 | 3 | 4 | 5 | |
| 10、您对本单位职业发展的机会满意吗? | 1 | 2 | 3 | 4 | 5 | |
| 11、你对在本单位获得荣誉和奖励的机会满意吗? | 1 | 2 | 3 | 4 | 5 | |

# 三、职业认同度

| **说明：**这部分问卷目的在于了解您对所从事职业的感知和正面评价的程度。请您根据自己的感受和体会在相应数字上打√。 | 完全不符合 | 不太符合 | 不能确定 | 比较符合 | 非常符合 |
| --- | --- | --- | --- | --- | --- |
| 1、当谈到我的职业时，我通常说“我们”，而不是“他们” | 1 | 2 | 3 | 4 | 5 |
| 2、医护工作者职业的成功就是我的成功 | 1 | 2 | 3 | 4 | 5 |
| 3、我很在乎别人对我职业的看法 | 1 | 2 | 3 | 4 | 5 |
| 4、别人赞美我的职业，就像是对我个人的赞美 | 1 | 2 | 3 | 4 | 5 |
| 5、如果有媒体批评我的职业，我会觉得很难堪 | 1 | 2 | 3 | 4 | 5 |
| 6、我的工作是重要的 | 1 | 2 | 3 | 4 | 5 |
| 7、我对自己的工作能力有信心 | 1 | 2 | 3 | 4 | 5 |
| 8、我做的工作影响患者状态 | 1 | 2 | 3 | 4 | 5 |
| 9、我的工作有意义 | 1 | 2 | 3 | 4 | 5 |
| 10、我具备了必要的业务能力 | 1 | 2 | 3 | 4 | 5 |
| 11、我了解工作的内容与要求 | 1 | 2 | 3 | 4 | 5 |
| 12、医疗工作适合我 | 1 | 2 | 3 | 4 | 5 |
| 13、我了解自己的角色 | 1 | 2 | 3 | 4 | 5 |

调查日期：2017年____月____日
